# Extrinsic Factors Shaping the Skin Microbiome

**DOI:** 10.3390/microorganisms8071023

**Published:** 2020-07-10

**Authors:** Veronica Moskovicz, Adi Gross, Boaz Mizrahi

**Affiliations:** Faculty of Biotechnology and Food Engineering, Technion, Haifa 3200003, Israel; veromoscovicz@gmail.com (V.M.); adigross@technion.ac.il (A.G.)

**Keywords:** microbiome, extrinsic influences, dysbiosis, skin, microbiome research, skin health

## Abstract

Human skin, our most environmentally exposed organ, is colonized by a vast array of microorganisms constituting its microbiome. These bacterial communities are crucial for the fulfillment of human physiological functions such as immune system modulation and epidermal development and differentiation. The structure of the human skin microbiome is established during the early life stages, starting even before birth, and continues to be modulated throughout the entire life cycle, by multiple host-related and environmental factors. This review focuses on extrinsic factors, ranging from cosmetics to the environment and antibacterial agents, as forces that impact the human skin microbiome and well-being. Assessing the impact of these factors on the skin microbiome will help elucidate the forces that shape the microbial populations we coexist with. Furthermore, we will gain additional insight into their tendency to stimulate a healthy environment or to increase the propensity for skin disorder development.

## 1. Introduction

We are not alone: Both inside and out, in tissues and biofluids, our bodies coexist with a huge array of microorganisms. The collection of microorganisms residing in the body, including bacteria, archaea, viruses, and eukaryotes, as well as their genomes and the surrounding environmental conditions, constitutes the microbiome [1,2]. The human microbiome is a complete and complex ecosystem that is organized in communities displaying great diversity according to body site, host age and sex, diet, genetics, socioeconomic status, geography, pregnancy status, and environmental exposure [3]. The distinctive microbial composition of the different body niches is related to their participation in the fulfillment of the host’s normal physiological functions. The arrangement of colonizing microbes is adapted to the particular environment and presents metagenomic elements to fit the requirements [4,5]. The gut and skin microbiome modulates the immune system. However, while the skin microbes participate in epidermal development and differentiation [6], the gut microbiome is involved in nutrient uptake and metabolism [7].

The assembly and equilibrium of the human microbiome are influenced by both host-related and external factors [8]. Daily life actions such as touching, eating, and breathing shape the human microbiome from its inception and can alter its composition throughout all life stages of an individual, by the same mechanisms. Even though the human microbiome shows some resistance to change and, to a certain extent, ability to recover its baseline composition after an alteration [4,5], powerful and selective forces can affect microbiome behavior and result in temporal or permanent alterations [9]. This is particularly evident in the skin, the human body’s largest organ and main interface [10,11]. The skin, being the most environmentally exposed surface, is continuously influenced by chemical, biological, and physical variables that can impact its stability and composition. Exposure to these variables may be temporary and short-lasting, as in the case of topical antiseptics and ointments, or repeated and long-lasting, such as ultraviolet (UV) radiation, fabrics, and cosmetics. In this manuscript, we review recent findings on the effect of extrinsic factors on the skin microbiome and discuss their repercussions on skin properties and health.

## 2. The Healthy Skin Microbiome

Human skin is acidic, cool, desiccated, and relatively sparse in nutrient availability compared with other organs [11,12], thus representing a less-than-ideal habitat to support bacterial growth. The presence of salt-rich sweat and antibacterial molecules imposes yet another obstacle [13]. Nevertheless, the skin offers many niches that differ in their physical and chemical properties, such as temperature, moisture, pH, and oxygen availability, creating multiple microenvironments [13,14]. Thanks to the skin’s display of distinct habitats and the adaptation processes microbes have successfully undergone, a wide range of microorganisms are able to find in the skin a stable ecological niche that offers appropriate growth conditions and the required nutrients. For example, the growth of *Propionibacterium* species is supported by lipid-rich skin surfaces, such as sebaceous face sites [12,15], while *S. aureus* is found in moisturized skin, higher in temperature and humidity, like the axillary vault and toe web [11]. The coevolution between host and microorganisms, individual or communities, is thought to have resulted in the establishment of mutualistic interactions, in which one or both members benefit from the presence of the other [16,17].

**Figure 1:**
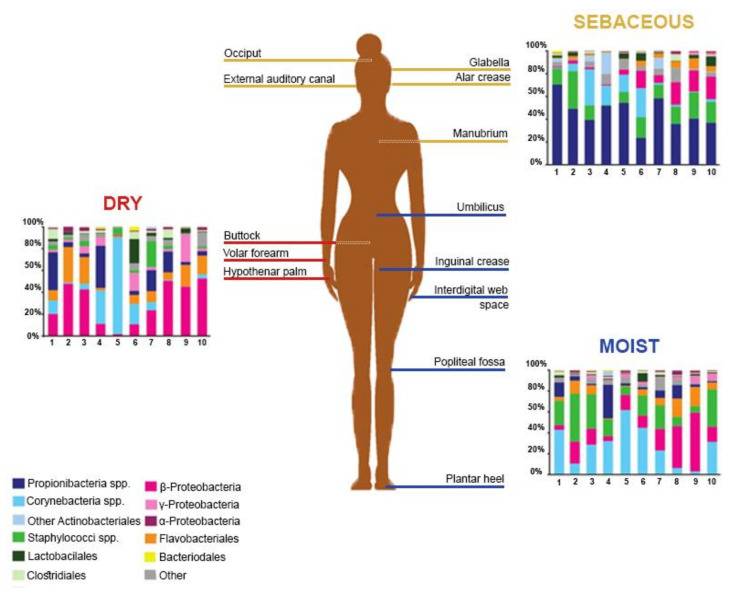
Topographical distribution of bacterial groups on various skin sites. The skin microbiome composition is highly dependent on the skin microenvironment. The bacterial composition of several sebaceous (yellow), dry (red), and moist (blue) skin sites of ten healthy human patients is shown. Data from Reference [15].

The Human Microbiome Project, initiated by the National Institutes of Health (NIH), gathered efforts to elucidate the healthy human skin microbiome [5]. Among the observations made, skin microbiome composition was found to be conserved at high taxonomic levels, while allowing more variance at lower taxonomic levels [12]. Members of the human microbiome belong to 19 bacterial phyla, the most represented being Actinobacteria, Firmicutes, Proteobacteria, and Bacteroidetes [16]. In terms of the skin microbiome, variations were observed between the proportions of bacterial species, probably resulting, among other factors, from technical differences in the targeted skin site, sampling methods, or sequencing strategies [16,17,18,19]. Griece et al. [15] characterized the topographical diversity of the human skin microbiome, sampling 20 body sites on ten healthy individuals associated with three microenvironments: sebaceous, moist, and dry (Figure 1). The proportions of the main skin-residing bacterial phyla were as follows: Actinobacteria at 51.8%, Firmicutes at 24.4%, Proteobacteria at 16.5%, and Bacteroidetes at 6.3%. Corynebacterium was the most represented genus on moist skin; Propionibacterium and Staphylococcus predominated on sebaceous skin; and β-Proteobacteria and Flavobacteriales were most abundant on dry skin. Gao et al. [19] assessed the composition of skin microbes taken from the superficial volar forearms of six healthy patients and found that bacteria belonging to Actinobacteria, Firmicutes, and Proteobacteria accounted for about 95% of the operational taxonomic units.

## 3. Microbiome Dysbiosis

The skin microbiome is a highly heterogeneous, yet organized and stable, assembly of microorganisms that form a complex network. When subjected to random short perturbations, the microbiome is able, to some extent, to remain undisturbed [4]. Continuous exposures to external pressures may, however, provoke destabilization of the equilibrium. This phenomenon, known as dysbiosis, describes an imbalance of the microorganism community on or within our body [20]. The high association between the bacteria that constitute the highly organized microbiome is thought to lead to a cascade effect, whereby a shift in one of the species leads to alterations in the others [4]. Such alterations can be of a diverse nature, ranging from an increased bacterium count, potentially replacing a previously lost function, or its complete depletion, possibly leading to a harmful condition. Dysbiosis has been widely studied in connection with several dermal diseases, such as atopic dermatitis (AD), acne, and vitiligo. In the case of AD, enrichment of *S. epidermidis* and of *S. aureus* is observed [21]. Given that *S. epidermidis*, a common member of the skin microbiome, is known to inhibit *S. aureus* growth [22], their coexistence in inflamed AD skin suggests an altered interaction that possibly influences disease progression. Some microbial dynamics theories attribute the observed microbial shifts in dysbiosis states to resource competition [23], nutritional interdependency leading to trophic cascades [24], or behavioral changes triggered by external or internal stimuli [25]. Nevertheless, the mechanisms of diversity alteration within microbial networks and the ensuing outcomes are highly complex and continue being investigated for each particular situation [26]. Whether dysbiosis is the cause of the disease or its outcome remains unclear. Naturally, current dysbiosis research compares individuals with established diseases with healthy subjects. The high variability in microbiome composition between individuals [17,27] theoretically requires researchers to compare the same individual before and after contracting a disease, in order to avoid bias and misleading results. This, however, would certainly have some ethical implications. Timing is yet another limiting factor for our ability to answer the question of what comes first; the width of the time window in which the microbiome can shift makes sampling time paramount. Despite these challenges, new therapeutic approaches to tackling skin dysbiosis are constantly being developed [28].

## 4. Strategies for Skin Microbiome Research

Advancements in the field of skin microbiome research depend on experimental performance, in which the selection of an appropriate biological model is crucial. Although several experimental systems have been proposed for the study of human skin microbiome, including 3D models [29], microfluidic co-culturing of eukaryotic cells and bacteria [30], and ex vivo tissue culture [31], the laboratory mouse is still the preferred model for the assessment of host–microbe interactions. The inner-ear skin of the mouse, for example, has a similar morphology and microbiome composition to that of human skin, and so that model has been used extensively for skin-barrier and immunity research [17].

**Figure 2:**
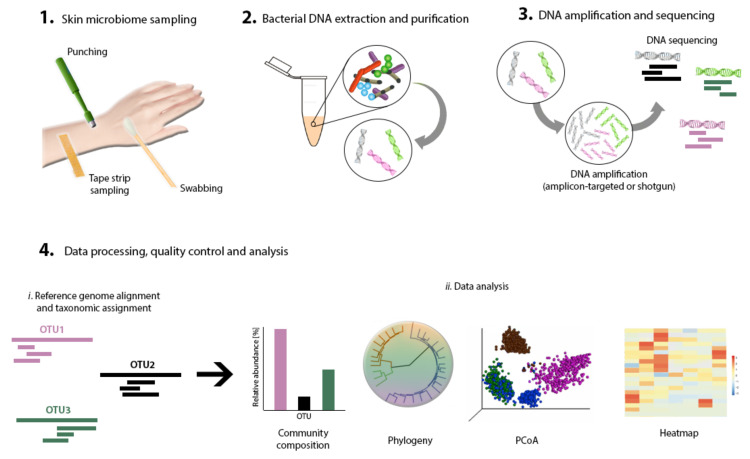
Skin microbiome research workflow. Primary steps include skin sampling, bacterial DNA extraction, purification, amplification and sequencing, data processing, and analysis.

Next comes sampling to capture a representative collection of bacteria residing in a specific skin area of interest. Swabs, biopsies, surface scrapes, and tape strips have been documented and validated as skin-sampling strategies [32] (Figure 2). Studies have shown high concordance between these sampling methods for the determination of skin microbiome composition by sequencing [30,33,34], although moistened swabbing is preferred for being non-invasive and easy to perform [35]. Ultimately, however, the method of choice should fit the research question. Consistent sampling of the anatomic area is of paramount importance in order to correctly compare samples and conduct well-grounded result interpretation.

Bacterial DNA extraction is required next, to capture the diversity of the microbial community present in the sample (Figure 2). Most extraction methods rely on the same basic steps: cell lysis, DNA purification, and collection [36]. Commercial, ready-to-use extraction and purification kits are available and have been tested for performance in microbiome research [33,37]. The acquisition of sufficient quantities of DNA from low-bioburden skin for the sequencing pipeline is not a trivial feat. Thus, to perform DNA extraction by using commercial kits and still obtain high DNA yield and quality [38], some modifications to the suggested protocols may be needed—for example, repeating elution steps and decreasing hands-on time to the bare minimum [33].

Until recently, microbiome research has relied almost exclusively on bacterial cultivation techniques [39]. However, since only less than 1% of the bacteria in a sample can grow under culture conditions, over 99% of the bacteria present in a sample had been overlooked [40]. The limitations of these methods accounted for the need to develop new strategies. Nowadays, DNA-based approaches are the preferred method for microbiome characterization (Figure 2). Developments in high-throughput DNA sequencing tools (next-generation sequencing), as well as the reduction of associated costs, have allowed the wide-ranging study of microbiomes in vivo [41]. Computational tools allowing increased capacity for big data processing have also simplified the performance of microbiome research [42]. DNA-based studies often fall into one of two categories: amplicon-targeted or shotgun metagenomics. While the former uses one or several marker genes, the latter targets the entire profile of gene content, to allow for the identification of the bacterial composition. The most suitable technique depends on the research question, since each method provides different metagenomics information. For amplicon-targeted microbiome studies, the 16S ribosomal DNA (16S rDNA) gene is the gene most commonly used [36,43]. This gene is a phylogenetic marker that is present in all living organisms [32]. It contains nine hypervariable regions (V1–V9) characterized by high sequence diversity between different bacteria [44], allowing their identification [43]. For skin microbiome research, which is characterized by a low bacterial biomass, targeting the V3–V4 region of the 16S rDNA gene results in good capture and representation of skin microbial communities [45]. In the case of shotgun metagenomics, random primers are employed to sequence overlapping regions of DNA and assemble whole genomes [36,46].

The amplification and sequencing of the extracted DNA enables us to determine the microbial structure of the skin microbiome. Many sequencing platforms that implement different DNA-sequence decoding technologies have been applied to genome and 16S sDNA amplicon sequencing [41]. These platforms include Roche 454 GS FLX, Illumina (MiSeq and HiSeq), Ion Torrent/IonProton/Ion Proton, SOLiD 5500 series, and Oxford Nanopore, to name a few. The density and complexity of the information contained on the microbiome makes the use of computational, statistical, and bioinformatics tools indispensable, in order to obtain a proper interpretation [47,48]. In most metagenomics projects, data analysis forms a bottleneck due to the massive datasets obtained [49]. A common data-analysis workflow includes data filtering and normalization, identification of microbial groups, classification and clustering, diversity analysis, and data visualization (Figure 2). Several software programs that accomplish this are now available [41]. For a complete, in-depth review article on this topic, refer to “Performing Skin Microbiome Research: A Method to the Madness” [32].

## 5. Extrinsic Influences Shaping the Skin Microbiome

Numerous sources of extrinsic factors exist that can shape the skin microbiome. Exposure can be either long- or short-term, continuous or one-time. The duration of the exposure significantly influences the effects on the microbiome [50]. Naturally, exposure intensity also plays an essential role in shaping the skin microbiome. Table 1 summarizes some of the extrinsic factors and their effect on skin microbiome.

### 5.1. Early Life Exposures

Bacterial colonization on the human skin begins at the moment of birth and continues developing and reshaping throughout our first years of life [70]. Reports on microbial colonization beginning before and during delivery have been thoroughly reviewed by others [71,72,73]. Krieger et al. [51] found fetal exposure to meconium-stained amniotic fluid (MSAF) in utero to be protective against the development of dermatitis and skin-eruption-related hospitalizations. Based on the widely investigated intimate gut–skin interaction, Krieger et al. [74] suggested that, through MSAF exposure, the diversity of the gut microbiome could be increased, stimulating the immune system and preventing later skin-inflammation-related diseases. The retrospective design of the study, however, limited the identification of the causes underlying these findings. Further research, including analysis of the bacterial profile of MSAF, is required.

Diaper dermatitis (DD) is an inflammatory skin condition caused by high-frequency abrasion between an infant’s skin and the diaper’s surface. The affected skin is highly susceptible to microbial infections, in particular to intestinal microbial residues [52]. The effect of diapers on skin microbiome was studied on 85 babies, of which 54 suffered from DD. Interestingly, bacterial diversity in DD patients was higher compared with the healthy controls [52], contradicting the notion that a greater bacterial diversity is advantageous [75]. Increased presence of enterococci, a normal skin and gut bacteria with pathogenic potential, was also found in DD lesions, supporting previous reports associating the bacterium to the development of DD [76]. On the other hand, Lactobacillus and Bifidobacterium, two gut probiotic bacteria, exhibited reduced levels in DD lesions [52]. The detection of intestinal bacteria on both healthy and DD skin led to speculations regarding a possible involvement of these microorganisms in skin ecology. Nevertheless, the authors express the need to deepen the research in order to determine whether a clear relationship exists.

### 5.2. Cosmetics

Skin hydration cosmetics are very popular among a large proportion of the population. They are used on a daily basis, to prevent dryness and maintain smooth and healthy skin [77]. The effect of hydration cosmetics on facial skin microbiome, specifically on the cheeks, was assessed by Lee et al. [53] on 30 volunteers with dry to hydrated skin types. The basic hydration set included four cosmetic products, which were applied sequentially after facial washing, twice a day, for four weeks: a skin softener, a lotion, an essence, and a cream. The use of these products resulted in a significant increase in bacterial diversity, regardless of facial-skin hydration level. As for the bacterial composition, the communities found in both skin types were different before and after cosmetic usage for all participants: Variability was related more to interpersonal differences than to skin hydration level. It is worth noting that the relative abundance of Propionibacterium, one of the most predominant skin bacterial genera, differed significantly between individuals with different skin hydration levels. Subjects with highly hydrated skin exhibited higher Propionibacterium abundance than those with low hydration. Furthermore, its abundance decreased significantly with cosmetic use, especially among the high-hydration group. Propionibacterium contains lipophilic skin commensals usually found in sebum-rich skin areas like the head, chest, and back [54]. This bacterial genus is reported to hydrolyze triglycerides present in human sebum as a nutrient source [78]. Cosmetic use promotes increased hydration levels, resulting in a drop in sebum content, which could possibly explain the decrease in Propionibacterium abundance. Among the Propionibacterium genus, *P. acnes* has been widely studied for its association with acne and immunomodulatory effects [54].

**Figure 3:**
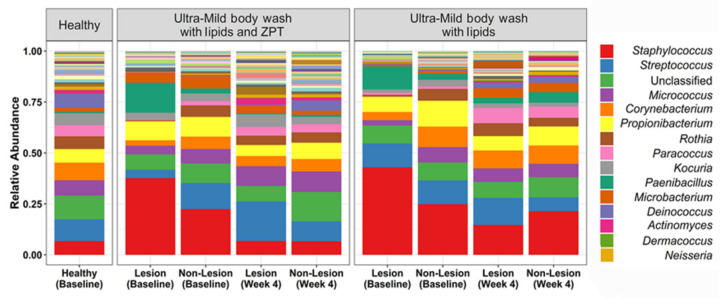
Bacterial relative abundance after treatment with lipidic body washes, with and without zinc pyrithione (ZPT), on lesional and non-lesional skin of young AD patients. The AD skin microbiome achieved a similar composition to the healthy controls after four weeks of body wash with lipids and ZPT treatment, and reduction in the relative abundance of *S. aureus*. Adapted from Reference [55].

The effect of lipidic body-wash formulas on the skin of young AD patients was assessed [68]. Atopic dermatitis affects up to 20% of children and 30% of adults worldwide, with patients experiencing cutaneous infections characterized by dry, red, and itchy skin [79]. As previously mentioned, a microbiome dysbiosis pattern characterized by an increased abundance of *S. aureus* is also distinctive of AD skin [21]. Lipidic body-wash formulas with and without zinc pyrithione (ZPT), a demonstrated active material against bacteria and fungi, were tested to analyze their capacity to improve AD skin condition. After body wash with lipids and zinc pyrithione treatment, bacterial composition was seen to shift toward that of the healthy controls. Both formulas resulted in increased microbial diversity, generally associated with healthy skin (Figure 3). However, only ZPT-containing lipid body wash significantly reduced Staphylococcus abundance at the genus level, and *S. aureus* at the species level, probably due to the deposition of this active material on skin surfaces and its subsequent interaction with bacterial cells to control their population. Nevertheless, assessment of SCORAD (SCORing of Atopic Dermatitis) scores revealed that AD severity remained unchanged in treated patients [68], supporting the theory that the impact of microbes in AD results from bacterial communities and their interactions, rather than from isolated bacteria [21,80,81]. 

### 5.3. Environment and Nature

The ecosystems humans live in, which are affected by the status of biodiversity, climate, and urbanization, among other factors, shape the human skin microbiome [82]. Van Mierlo et al. [56] investigated the influence of the alpine climate, characterized by lower pollution and allergen levels and increased UV radiation, on lesional and non-lesional skin of children with difficult-to-treat AD. After six weeks of alpine climate treatment, a significant shift in the general skin microbiome of AD lesions was observed. In particular, a decrease was seen in *S. aureus* abundance on diseased skin after the treatment. High *S. aureus* abundance has been associated with increased disease severity in AD patients [83], suggesting that an alpine climate could assist in AD treatment. Nevertheless, the authors show uncertainty when determining whether the observed shifts in *S. aureus* abundance cause or are caused by changes in disease severity [56].

Ultraviolet radiation (UV-R) induces synthesis of antimicrobial peptides (AMPs), essential components of the innate immune system that trigger defense responses. However, UV-R is also associated with skin cancer development and immune suppression, as well as photoallergic and phototoxic responses [84]. The role of the microbiome in the UV-R–immune system interaction has not been investigated until recently. Patra et al. [57] demonstrated that the microbiome acts as an intermediate between UV-B radiation exposure and the skin’s immune response by using germ-free (GF) mice (completely lacking microbiomes) and comparing them with specific-pathogen-free (SPF) mice. UV-B exposure led to a pro-inflammatory environment in the microbiome-holder SPF mice, whereas an immunosuppression reaction was favored by the microbiome-free GF mice [57]. This was evidenced by the differential gene expression observed for both animal groups, as a response to UV-B exposure: Pro-inflammatory cytokines (IL-1β, IL-6, and IL-18rap) were predominant in the presence of a microbiome, while immunosuppressive cytokines (IL-10, IL-10ra, IL-20rb, and IL-7r) were found in the absence of a microbiome. Based on these findings, the authors suggested that the microbiome has a protective effect against immune suppression caused by UV-B.

Soils and plant leaves are major microbe sources. Vandegrift et al. were first to examine the effect of human–soil and human–leaf contact on the skin microbiome [58]. The authors, who hypothesized that soil and plant leaves could cause transient compositional changes in the skin microbiome, showed that the movement of microbes from the donor soil or leaf sample to the human receptor caused a diversity alteration in the subject’s skin microbiome. This alteration was transient and characterized by a bacterial composition resembling that of the donor, both for leaves and soil samples. The time required to return to the basal state was found to depend on the initial biomass in the source communities. The most abundant taxa in the donor source were expected to have a higher probability of colonization in the human receptor. This hypothesis was supported by the observation that only a small amount of highly represented taxa coming from the donor microbiome remained after 24 h and survived a thorough wash of the human receptor [58].

### 5.4. Antimicrobial Agents

Antimicrobial agents are used with the intent of avoiding or reducing infections caused by microorganisms. Increasing evidence of the relevance of the microbiome for human physiology and health has, however, led to a paradigm shift: Microbes and bacterial colonization are no longer perceived strictly as threats, but rather depending on the circumstances. As a result, the effects of the intake of non-specific antimicrobials on resident bacterial communities are now being reconsidered since they also affect beneficial, commensal members. As an example of the commensal–pathogenic bacteria duality, *S. epidermidis* and *C. acnes* have been indicated as acne-causing agents [85] and, conversely, as resident bacteria that inhibit pathogenic bacteria growth and decrease the risk of disease development. Nevertheless, current treatment options for acne include antibiotics, and comedolytic and anti-inflammatory agents designed to attack the bacterial component of the disease [86]. In a recent research study, the skin microbial composition of individuals suffering from acne was analyzed before and after four weeks of oral minocycline administration [59]. Antibiotic administration caused a decrease in the variety of bacterial species of the skin: relative abundances of Cutibacterium, Corynebacterium, Prevotella, Lactobacillus, and Porphyromonas decreased, and only Porphyromonas was able to recover its baseline levels eight weeks after discontinuation of minocycline treatment. These results support the hypothesis that systemic antibiotic treatment may negatively impact skin health by reducing the abundance of protective bacteria, such as Lactobacillus, which is known to suppress *S. aureus* infections [87,88,89], atopic dermatitis [90], and acne [90]. New microbiome-based interventions, which favor the growth of commensal bacteria rather than attack pathogens, are under investigation for the treatment of skin conditions; examples include virus-derived endolysins for the treatment of staphylococcal impetigo [91] and coal tar therapy for the alleviation of AD symptoms [92].

**Figure 4:**
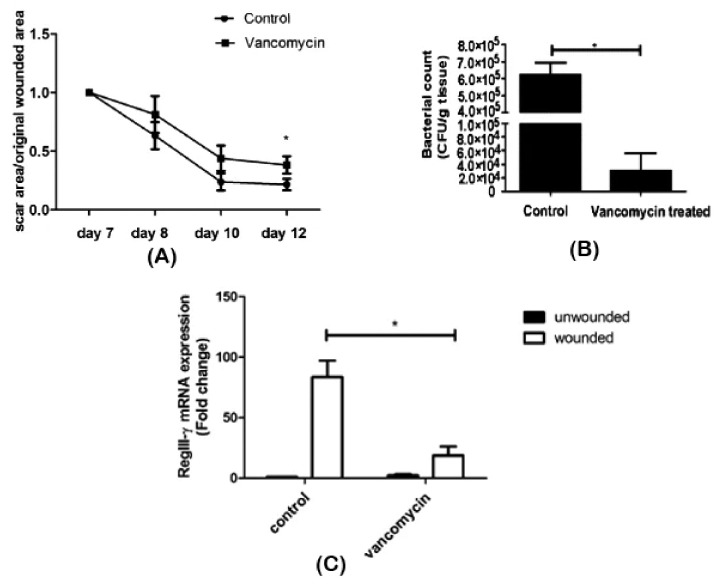
Impaired wound healing after vancomycin therapy. (**A**) Delayed wound repair was observed, (**B**) associated with reduced bacterial density and diversity and (**C**) decreased RegIII-γ expression in scar tissue. *p < 0.05. Adapted from Reference [93].

Zhang et al. [60] assessed the effect of oral vancomycin treatment on wound repair and its association with the skin microbiome. After seven days of vancomycin administration, an excisional wound was created on the back of shaved mice and monitored for five days. Bacterial enumeration was performed on scar tissue, and RNA was isolated from wounded and unwounded skin samples. Antibiotic intake was seen to have a delaying effect on scar formation (Figure 4A). Alteration of the bacterial composition on scar tissue was observed, as well: While Staphylococcaceae was dominant in control mice, a decrease in its abundance and Lactobacillaceae predominance was found in vancomycin-treated mice (Figure 4B). Surprisingly, as observed in the RNA profiling, vancomycin treatment resulted in an underexpression of RegIII-γ (Figure 4C), a gene expressed in wound tissues to induce keratinocyte proliferation and promote healing [93]. Although the implication of the Lactobacillaceae family in wound healing still needs to be elucidated, the authors were able to conclude that antibiotic therapy caused a microbiome shift and delayed wound healing.

Dellacecca et al. [61] studied the influence of oral ampicillin treatment on the microbiome of vitiligo skin. Upon antibiotic administration, treated animals were monitored for skin depigmentation. Both skin and fecal microbiomes were analyzed. Ampicillin intake was shown to cause a 2.4-fold acceleration in skin depigmentation, compared with control animals. While untreated mice did not experience pelage pigment loss, mice under antibiotic treatment presented with 33% depigmentation at week 30. Cutaneous microbiome analysis revealed no impact of oral antibiotic treatment on microbial colonization, but the gut microbiome was highly affected, as evidenced by a significant drop in bacterial abundance. A clear association was, therefore, observed between the distant gut microbiome composition and vitiligo development. The authors suggest that antibiotic treatment may stimulate pro-inflammatory and antigen-presenting gut bacterial strains, leading to the activation of T cells against melanocytes and causing depigmentation [61]. 

Ozone has been used for more than a century for its ability to inactivate a wide range of microorganisms, stimulate oxygen metabolism, and activate the immune system [94]. Zeng et al. studied the effect of topical ozone on the microbiome diversity of AD skin by submitting patients to ozonated water showers, followed by application of topical ozonated oil [62]. *S. aureus*, whose colonization is highly associated with AD, was present in 58% of the skin lesions. Ozone therapy significantly mitigated the AD lesions; this observation was associated with increased microbiome diversity and compositional changes in the bacterial populations of AD lesions. Staphylococcus abundance decreased as a result of the ozone treatment and was found to be partially correlated with disease severity. A decrease was observed in the abundances of Acinetobacter, Lactobacillus, Streptococcus, and Propionibacterium, as well. The authors suggest that short-term ozone therapy can enhance AD treatment by restoring microbial diversity, exhibiting antibacterial properties to reduce Staphylococcus abundance, repairing skin barrier function, and relieving inflammatory reactions [62].

### 5.5. Bacterium-Based Agents

Probiotics, introduced in the 1900s, thanks to their positive influence on human health [95], were defined by the FAO with support from the WHO as “live microorganisms which when administered in adequate amounts confer a health benefit on the host” [96]. The use of probiotics has been proven to reduce infection frequency and antibiotics usage. Particularly, researchers have demonstrated their efficacy in the treatment of skin conditions such as AD and acne vulgaris, potentially due to their contribution to the inhibition of skin pathogen growth [97] and promotion of a positive bacterial balance [98]. Bacterial probiotic genera for the skin include Lactobacilli and Bifidobacterium [98]. Probiotics, which are not only efficient for the treatment of skin conditions, are also used in general skin healthcare, to improve the skin’s physicochemical properties (hydration, elasticity, melanin production, etc.) and immune system [99,100,101]. In AD, *L. rhamnosus* successfully reduced the risk of disease development in children [63] and the severity of eczema in 56% of pediatric patients when combined with *L. reuteri* [64]. No action mechanism was suggested to account for the healing properties other than the possible reestablishment of a healthy bacterial balance [99]. In another study, a mixture of *L. acidophilus*, *L. delbrueckii,* and *B. bifidum* was tested as a probiotic supplementation for the treatment of acne [65]. A significant decrease in lesion number was observed, although the force behind the healing process was not fully elucidated. In an investigation of wound healing, *L. acidophilus* and *L. casei* efficiently reduced methicillin-resistant *S. aureus* (MRSA) counts in vitro, suggesting a potential decline in the risk of infection [66]; however, no in vivo experiments were conducted. In vivo testing is crucial for microbiome research, since in vitro conditions are optimal and far from representing real tissue conditions. The first clinical case of a topical probiotic formulation designed to treat chronic ischemic wounds infected by three multi-drug-resistant bacteria, *Klebsiella pneumoniae*, *Enterococcus faecalis,* and *Proteus mirabilis*, was recently reported [67]. The rescue probiotic treatment consisting of *Lactobacillus plantarum*, *Lactobacillus acidophilus,* and *Streptococcus thermophiles* achieved complete wound healing after 24 days, whereas antibiotic-based treatments failed. The healing mechanism was attributed to the inhibition of pathogen growth and modulation of host immune responses led by the probiotic bacteria [67]. All of the above mentioned are oral probiotics, representing an indirect method of skin microbiome alteration for the benefit of human health. The availability of topical probiotics is limited, but research demonstrates favorable results. In this context, the concept of a skin bacterial transplant (SBT), which mimics the contemporary gut bacterial transplant, was recently tested. SBT refers to the transplantation of diverse cutaneous microbes from one individual to another, to modify the bacterial composition and promote health [102]. Many studies focus on the transplantation of human-derived isolated bacterial species. For example, the skin commensal *S. hominis* has been successfully transplanted to AD skin, for its ability to produce AMPs (such as cathelicidins and β-defensins), inhibiting *S. aureus* growth and improving disease symptoms [68]. A phase I/II human trial was recently conducted to test the use of *R. mucosa* isolated from healthy volunteers as a potential AD treatment [69]. Results showed a significant decrease in disease severity, accompanied by a decrease in *S. aureus* colonization and topical corticosteroid use. One recent clinical trial used a whole microbiome transplantation for the treatment of AD; however, these results have not yet been published [103]. To the best of our knowledge, current studies assessing bacteriotherapy (any use of bacteria or bacterial components for therapeutic benefits) all focus on comprehending the bacterial effect on skin conditions, but are not microbiome oriented. This means that they do not always consider the effects of adding external bacteria to human skin bacterial communities. Investigating this aspect could lead to a better understanding of how external bacteria, including probiotics, components of the normal microbiome or others, influence the composition of the microbiome and their connection to skin health and disease.

## 6. Future Perspectives

The field of microbiome is undergoing intense study, integrating knowledge from various fields. The complex characteristics of the microbiome, combined with the imminent interactions between the microbial ecosystems and the host, make the complete elucidation of the human microbiome a complicated task. The NIH’s Human Microbiome Project has risen to the challenge, gathering interdisciplinary efforts to elucidate the mechanisms behind the microbiome and its impact on human health and disease, as well as to provide resources to promote progress in the area [104]. However, there is still a long way to go in order to fully elucidate the human microbiome. In particular, the effect of extrinsic, non-host dependent factors on the microbiome must be addressed in order to enable the modulation of the skin microbiome for the benefit of human health. Future research should focus on assessing the effect of extrinsic factors on a healthy skin microbiome, taking into consideration length and intensity of exposure, and subsequently characterizing their effect on diseased skin. By combining the information, a clearer picture may be obtained of the implications of extrinsic factors for skin disease development or prevention through microbiome modulation.

## Figures and Tables

**Table 1:** Summary of extrinsic factors affecting the skin microbiome.

Classification	Factor	Skin Condition	Effect on Microbiome	Clinical Outcome	Reference
Early life exposures	Meconium-stained amniotic fluid	Healthy	↑ gut microbiome diversity	Prevention of skin inflammation-related diseases	[51]
	Diapers	Diaper dermatitis	↑ *Enterococcus* and *S. aureus* abundances	Not described	[52]
			↓ *Lactobacillus* and *Bifidobacterium* abundances		
Cosmetics	Skin hydration set	Healthy	↑ bacterial diversity	Not described	[53]
			↓ *Propionibacterium* abundance		
	Madecassoside	Healthy	↓ *P. acnes*	Enhanced skin hydration	[54]
				Suppressed inflammation	
	Lipidic body wash with ZPT	Atopic dermatitis	Bacterial shift to healthy controls	No effect on AD severity	[55]
			↓ *S. aureus* abundance		
Environment and nature	Alpine climate	Atopic dermatitis	↓ *S. aureus* abundance	Decrease in disease severity	[56]
	UV-B radiation	Healthy	Immune response intermediate	Protection against immuno-suppression	[57]
	Soil and plant leaves	Healthy	Altered microbiome diversity	Transient bacterial shift dependent on donor bacterial biomass	[58]
			Resemblance of human bacterial composition to that of the donor (soil/leaf)		
Antimicrobial agents	Minocycline	Acne	↓ *Cutibacterium*, *Corynebacterium*, *Prevotella*, *Lactobacillus,* and *Porphyromonas*	Reduced abundance of skin-protective bacteria	[59]
	Vancomycin	Wounded skin	↓ Staphylococcaceae	Delayed wound repair	[60]
			↑ Lactobacillaceae		
			↓ RegIII-γ		
	Ampicillin	Vitiligo	↓ gut bacterial abundance	Accelerated depigmentation	[61]
	Ozone	AD	↑ skin microbiome diversity	Mitigation of AD lesions	[62]
			↓ *Staphylococcus, Acinetobacter, Lactobacillus, Streptococcus,* and *Propionibacterium* abundances		
Bacterium-based agents	*L. rhamnosus*	AD	No mechanism described	Reduced risk of disease development in children	[63]
	*L. rhamnosus* and *L. reuteri*	AD	No mechanism described	Reduced disease severity in children	[64]
	*L. acidophilus*, *L. delbrueckii,* and *B. bifidum*	Acne	No mechanism described	Reduced lesion number	[65]
	*L. acidophilus* and *L casei*	Wounded skin	↓MRSA	Reduced risk of infection	[66]
	*K. pneumoniae, E. faecalis,* and *P. mirabilis*	Chronic ischemic wounds	↓Pathogen growth	Complete wound healing	[67]
			Immune response modulation		
	*S. hominis*	AD	↓*S. aureus* abundance	Improved disease symptoms	[68]
	*R. mucosa*	AD	↓*S. aureus* abundance	Reduced disease severity	[69]
